# Emotional processing deficits mediate the association between adverse childhood experiences and CPTSD symptoms: the moderating role of perceived social support

**DOI:** 10.3389/fpsyt.2026.1834482

**Published:** 2026-04-28

**Authors:** Chi Zhang, Dong Zhai, Qian Wang

**Affiliations:** 1 Psychological Quality Education Center, Beijing Jiaotong University, Beijing, China; 2 School of Marxism, Beijing Jiaotong University, Beijing, China; 3 Department of Psychology, School of Humanities, Tongji University, Shanghai, China

**Keywords:** adverse childhood experiences, CPTSD, emotional processing deficits, moderated mediation, perceived social support

## Abstract

**Background:**

Adverse childhood experiences (ACEs) are robust predictors of adult psychopathology and have been strongly associated with complex post-traumatic stress disorder (CPTSD). While the association between ACEs and CPTSD is well established, the specific psychological mechanisms and external interpersonal protective factors shaping this developmental association remain insufficiently understood.

**Methods:**

This study examined the mediating role of emotional processing deficits (EPD) and the moderating role of perceived social support (PSSS) in the associations between early cumulative adversity and adult CPTSD dimensions. Data were collected from a non-clinical sample of 5, 771 Chinese university students using a cross-sectional survey. A latent moderated structural equation modeling (LMS) approach was employed to examine the proposed relationships.

**Results:**

Results indicated that emotional processing deficits significantly mediated the positive association between cumulative childhood adversity and both the core PTSD and Disturbances in Self-Organization (DSO) symptom clusters. Furthermore, perceived social support served as a modest but statistically significant moderator in the second stage of the mediation process; high levels of interpersonal support partially attenuated the positive association between EPD and subclinical CPTSD symptoms. Finally, while cumulative adversity showed a statistically stronger unstandardized association with DSO than with core PTSD symptoms, the standardized effect sizes were comparable (β = 0.151 vs. 0.152). This indicates that while there is a statistically significant absolute difference on the psychometric scale, the relative magnitude of the associations is parallel across both CPTSD dimensions.

**Conclusions:**

These findings highlight emotional processing deficits as a pivotal correlate linking early adversity to CPTSD symptoms. Importantly, the protective efficacy of social support is not universal; while interpersonal resources may partially buffer the association between internal psychological vulnerabilities and subclinical symptoms, this effect is contingent upon the individual’s trauma history and the qualitative source of support, with peer networks potentially offering unique compensatory value in trauma recovery.

## 1 Introduction

Adverse childhood experiences (ACEs) represent a significant and pervasive global public health challenge, encompassing diverse forms of maltreatment, neglect, and various modes of household dysfunction occurring before the age of 18 (1–3). Accumulated evidence indicates that the deleterious effects of ACEs exert profound cross-lifespan consequences, altering neurobiological development and serving as robust predictors of impaired physical health and a wide spectrum of psychopathology in adulthood (4–6). Recent three-level meta-analytic findings further emphasize that cumulative exposure to ACEs is significantly more relatively associated with Complex Post-Traumatic Stress Disorder (CPTSD) than with traditional PTSD, particularly when trauma is chronic and interpersonal in nature—a relationship that suggests a clear dose-response effect (7–9). With the formal introduction of CPTSD in the 11th revision of the International Classification of Diseases (ICD-11), clinical attention has shifted from a narrow fear-based construct to a broader framework (10–13). This new framework incorporates “Disturbances in Self-Organization” (DSO), which encompasses pervasive dysregulation in affect, negative self-concept, and relational functioning (14, 15). Despite the well-documented link between ACEs and CPTSD, there is a lack of systematic evidence regarding how internal psychological mechanisms and external social resources interact to shape this developmental association, particularly among young adults undergoing critical transitions (16).

According to the ICD-11 conceptualization, CPTSD comprises the core PTSD symptom cluster (re-experiencing, avoidance, and hyperarousal) and the DSO symptom cluster (affect dysregulation, negative self-concept, and interpersonal disturbances; 17–19). Recent epidemiological studies have robustly validated the ICD-11 CPTSD construct within Chinese populations, highlighting its high prevalence and distinct clinical utility across cohorts (20–23). Despite this distinct dual-cluster structure, many studies continue to treat CPTSD as a unitary construct, which may obscure the differential pathways through which early trauma affects PTSD versus DSO dimensions (24). As emphasized by recent reviews, identifying the specific mediators of the DSO symptom cluster is a current research priority, as the remediation of DSO typically requires a deeper restructuring of self-regulatory mechanisms compared to core PTSD symptoms (25, 26).

Previous research has begun to explore psychological mechanisms linking ACEs to CPTSD symptoms (27). For instance, Guo et al. (28) demonstrated that self-compassion components, including self-kindness and self-judgement, mediated the association between ACEs and CPTSD symptoms among Chinese college students (28, 29). While these findings highlight the crucial role of internal self-evaluative processes, less attention has been paid to fundamental emotion processing capacities, which represent a more proximal, lower-level dimension of psychological functioning following early trauma.

Within this reparative framework, emotional processing deficits (EPD) are hypothesized to act as a central internal hub linking ACEs and CPTSD. It is crucial to distinguish EPD from emotion regulation (ER) (30). While ER primarily involves the strategies and goal-directed processes used to modify emotional intensity or expression (31, 32), emotional processing refers to the more fundamental capacity to identify, experience, and adaptively integrate emotional responses. According to the framework underlying the Emotional Processing Scale (EPS-15; 33), EPD manifests across distinct facets, including emotional suppression, unregulated emotional outbursts, impoverished emotional experience, signs of unprocessed emotion, and experiential avoidance. Chronic stress during critical developmental windows frequently overwhelms maturing neurobiological systems, leading to severe limitations in these foundational processing capacities (34, 35). Importantly, unlike alexithymia, which primarily denotes a stable trait-like inability to identify and describe feelings, EPD encompasses a broader spectrum of active and passive processing failures, including unregulated emotional outbursts and experiential avoidance. Consequently, individuals with high EPD may become trapped in affective instability and avoidance. This processing deficit not only impedes the adaptive integration of traumatic memories—a core driver of PTSD—but also directly erodes self-efficacy, ultimately manifesting as the pervasive negative self-cognitions characteristic of the DSO dimension (36, 37). Moreover, recent literature continuously highlights that the internal vulnerability caused by childhood trauma to some extent predicts adverse clinical outcomes, emphasizing the need to explore internal mediating mechanisms (38). Therefore, elucidating how fundamental emotional processing is disrupted following ACEs is crucial for understanding the endogenous origins of CPTSD symptoms.

However, the trajectory from early adversity to adult pathology is not deterministic; an individual’s psychological resilience typically hinges on the availability of compensatory resources (39, 40). The “stress-buffering hypothesis” posits that external social resources can effectively intervene and disrupt the pathway from internal psychological deficits to clinical symptoms (41–43). Perceived social support (PSSS) acts as a critical “external bypass moderator” within the pathogenic model of CPTSD (44). However, previous studies have reported mixed findings regarding the general efficacy of this buffering mechanism, including evidence for buffering effects, null or weakened effects, and, in some cases, reverse-buffering effects (e.g., 45, 46). This underscores the necessity of exploring the boundary conditions of social support, such as its specific sources and the individual’s trauma history. Therefore, clarifying these inconsistencies is critical for advancing theoretical understanding of the role of social support in trauma-related psychopathology. From an interpersonal neurobiology perspective, when an individual’s internal regulatory system is functionally paralyzed by early trauma, stable social support provides a scaffold for “co-regulation, “ assisting the individual in maintaining physiological and emotional homeostasis under stress (44, 47). This external scaffolding—provided through emotional validation, social belonging, and safety—compensates for internal functional deficits, thereby intercepting the symptom cascade triggered by emotional processing failures.

The rationale for positioning PSSS as a moderator in the second stage (EPD → CPTSD) rather than the first stage (ACEs → EPD) is rooted in the temporal nature of the variables. ACEs capture retrospective, distal events that occurred before age 18, whereas both PSSS (current college social networks) and EPD represent proximal, current psychological states. Conceptually, current social support cannot retroactively modify developmental neurobiological alterations associated with childhood adversity. Instead, its protective function manifests concurrently, intervening when current emotional processing deficits threaten to cascade into active CPTSD symptoms. This perspective aligns with socio-ecological models emphasizing the protective role of interpersonal resources in stress adaptation (42).

Building on this background, the present study utilizes a large sample of 5, 771 Chinese university students to test a moderated mediation model using latent moderated structural equation modeling (LMS). Specifically, we examine whether emotional processing deficits mediate the associations between ACEs and the dual dimensions of CPTSD (PTSD and DSO), and whether perceived social support functions as a protective buffer in this process. The present study focuses on the moderating role of perceived social support in the second stage of the mediation process (EPD → CPTSD). This specification is grounded in the temporal proximity of the constructs, as both emotional processing deficits and perceived social support reflect current psychological and interpersonal functioning in emerging adulthood. Accordingly, social support is conceptualized as a concurrent buffering resource that operates when emotional processing deficits translate into active symptom expression.

Based on these theoretical considerations, we propose the following hypotheses:

H1: Cumulative ACEs will be positively associated with both PTSD and DSO symptoms, with a potentially stronger unstandardized association with the DSO dimension.
H2: Emotional processing deficits (EPD) will act as a significant positive mediator in the relationship between ACEs and both PTSD and DSO symptoms.
H3: Perceived social support (PSSS) will significantly moderate the direct paths from EPD to PTSD and DSO symptoms; specifically, these positive associations will be attenuated under high levels of PSSS.
H4: PSSS will moderate the entire mediation pathway (moderated mediation), such that the indirect pathogenic effects of ACEs on PTSD and DSO via EPD will be buffered/attenuated when social support is high.

## 2 Methods

### 2.1 Participants and procedure

This study employed a cross-sectional design. Data were collected via an online survey platform administered to university students in Beijing, China. All participants provided electronic informed consent prior to assessment. To minimize social desirability bias, the survey was conducted anonymously, and participants were assured of strict data confidentiality. The study protocol was approved by the Institutional Review Board of the Institute of Psychology, Chinese Academy of Sciences (Approval No. H25031). A total of 5, 924 questionnaires were returned. During data cleaning, invalid responses (e.g., incomplete or patterned responses) and extreme cases (response times beyond ±3 SD of the mean) were strictly excluded. The final valid sample consisted of 5, 771 participants (valid response rate: 97.4%). Among them, 3, 325 (57.6%) were male and 2, 446 (42.4%) were female. Regarding residence, 4, 056 (70.3%) lived in urban areas. The vast majority (97.5%) reported no prior history of physical or mental illness.

### 2.2 Measures

#### 2.2.1 Adverse childhood experiences-international questionnaire

The Chinese version of the ACE-IQ (48, 49) was used to assess exposure to 13 categories of early adversity. Following the standard frequency scoring approach, item responses were dichotomized (0 = unexposed, 1 = exposed) for each category based on defined frequency thresholds. A cumulative risk score was then calculated by summing the exposures across all 13 categories (range: 0–13), which was included as an observed variable in the structural equation model. Internal consistency in the present study was acceptable (Cronbach’s *α* = 0.73).

#### 2.2.2 International trauma questionnaire

The ITQ (17) assessed symptoms of PTSD and CPTSD according to ICD-11 diagnostic criteria. Items 1–6 measure the three PTSD clusters (re-experiencing, avoidance, hyperarousal); items 10–15 measure the three DSO clusters (affect dysregulation, negative self-concept, interpersonal disturbances). ICD-11 diagnostic algorithms were strictly applied for prevalence estimation. In the SEM analysis, the three PTSD cluster scores and the three DSO cluster scores were utilized as observed indicators for their respective latent variables. The total scale exhibited excellent reliability (Cronbach’s *α* = 0.97 in the present sample).

#### 2.2.3 Emotional processing scale-15

The 15-item short form of the Emotional Processing Scale (EPS-15; 33) was utilized to assess the central mediating construct of emotional processing deficits (EPD). The EPS-15 evaluates functional deficits across five distinct dimensions: suppression, unregulated emotion, impoverished emotional experience, signs of unprocessed emotion, and avoidance. While higher total scores (range: 0–135) reflect greater overall impairment, the present study utilized the five specific subscale scores as observed indicators to construct the latent “EPD” variable in the structural equation modeling (SEM) analysis. This measurement approach effectively captures the multidimensional nature of emotional processing failures. The scale demonstrated excellent internal consistency in the current sample (Cronbach’s α = 0.94).

#### 2.2.4 Perceived social support scale

The Perceived Social Support Scale (PSSS; 50) was utilized to evaluate participants’ perceived support. Recent psychometric evaluations have demonstrated that the Chinese version of the PSSS exhibits excellent reliability and structural validity among Chinese populations facing high-stress circumstances (51). The scale assesses support across three distinct sources: family, friends, and significant others. The three subscale scores served as indicators for the latent “PSSS” construct, representing the strength of external buffering resources (Cronbach’s *α* = 0.98).

### 2.3 Covariates

To isolate the specific effects of the core research variables, participants’ demographic characteristics (gender, age, and subjective socioeconomic status) were included as covariates in all structural models.

### 2.4 Data analysis

Preliminary analyses were conducted using SPSS 26.0 (52). Harman’s single-factor test indicated that the first unrotated factor accounted for 28.48% of the variance, well below the 40% threshold, suggesting common method bias was not a primary driver of the results, though we acknowledge the inherent limitations of this test (53). More advanced procedural or statistical remedies are recommended in future research. Structural equation modeling (SEM) was conducted using Mplus 8.3 (54). Preliminary analyses revealed that the symptom outcomes exhibited severe zero-inflation floor effects and marked positive skewness and kurtosis (e.g., skewness range: 3.67–5.11; kurtosis range: 13.68–27.34). Thus, the robust maximum likelihood estimator (MLR) was utilized to obtain robust parameter estimates under non-normal distributions (55, 56). A confirmatory factor analysis (CFA) first evaluated the measurement model.

To test the hypothesized model involving a latent interaction (EPD x PSSS), the Latent Moderated Structural Equations (LMS) approach was employed (57) via a two-step procedure (58, 59). First, a baseline main-effects model (Model 0) was estimated to evaluate overall structural fit. Hypothesized path differences (H1) were examined using a Wald test. In Step 2, the full LMS model (Model 1) including the latent interaction was estimated. A log-likelihood ratio test (LLRT) indicated that Model 1 significantly improved model fit relative to Model 0 (Δ-2LL = 8.16, *p* <.05). Conditional indirect effects and the index of moderated mediation were computed to interpret specific buffering effects (60). To facilitate interpretation of the interaction effects, conditional effects of EPD on PTSD and DSO were estimated at low (−1 *SD*), mean, and high (+1 *SD*) levels of perceived social support.

Finally, to address potential estimation biases arising from the zero-inflated distribution of symptoms and to rigorously test the boundary conditions of the stress-buffering hypothesis, we conducted a series of sensitivity and exploratory analyses. We restricted the sample to individuals who explicitly reported experiencing a traumatic event on the International Trauma Questionnaire (ITQ; *n* = 1, 643) and re-estimated the general moderation model. Furthermore, we tested source-specific moderations (Family vs. Friends vs. Significant Others) across both the full and subsample to unpack the complexity of social support.

## 3 Results

### 3.1 Preliminary analyses and prevalence rates

Descriptive statistics and bivariate correlations are presented in **Table 1**. Cumulative ACEs were significantly and positively correlated with EPD, PTSD, and DSO, and negatively correlated with PSSS (all *p* <.001). Based on ICD-11 diagnostic algorithms applied to the ITQ, the outcome distributions were highly skewed, with 71.5% of participants reporting neither trauma exposure nor clinically relevant symptoms. The prevalence rate was 0.4% for PTSD and 1.2% for CPTSD, reflecting the non-clinical nature of the university sample.

**Table 1:** Descriptive statistics and bivariate correlations among main variables (*N* = 5, 771).

Variable	*M*	*SD*	α	1	2	3	4	5
1. ACEs	4.83	1.19	0.73	–				
2. EPD	29.31	27.67	0.94	0.585***	–			
3. PSSS	67.82	13.77	0.98	-0.441***	-0.444***	–		
4. PTSD	0.54	2.14	0.97	0.358***	0.309***	-0.182***	–	
5. DSO	0.77	2.57	0.97	0.385***	0.352***	-0.216***	0.825***	–

ACEs, Adverse Childhood Experiences; EPD, Emotional Processing Deficits; PSSS, Perceived Social Support; PTSD, Post-Traumatic Stress Disorder; DSO, Disturbances in Self-Organization. ***p <.001.

### 3.2 Measurement model

The CFA model demonstrated an excellent fit to the data: *χ²*(71) = 404.78, *p* <.001; CFI = 0.983; *TLI* = 0.978; *RMSEA* = 0.029 (90% CI [0.026, 0.031]); *SRMR* = 0.024. All standardized factor loadings were significant (range: 0.75–0.98, *p* <.001). Composite reliability (CR) values ranged from 0.86 to 0.96, exceeding recommended thresholds, and discriminant validity was established as the square root of the AVE for each construct exceeded its inter-construct correlations (61).

### 3.3 Structural model and moderated mediation

In the first step of the LMS procedure, the baseline main-effects model showed acceptable fit: χ²(116) = 1356.56, *p* <.001; CFI = 0.955; TLI = 0.942; RMSEA = 0.043; *SRMR* = 0.124. Although *SRMR* exceeded the conventional threshold, simulation studies have noted that *SRMR* can be inflated in models containing multiple observed exogenous variables and highly skewed outcomes. Similar patterns have been reported in latent interaction models estimated using *LMS* procedures, and therefore *SRMR* should not be interpreted in isolation when latent interaction models are estimated (59). In the second step, the full *LMS* model (including the EPD x PSSS interaction) was estimated. The LLRT indicated that adding the latent interaction significantly improved model fit (Δ-2LL = 8.16, *p* <.05), with a corresponding decrease in AIC. Overall, the full model accounted for 24.5% of the variance in PTSD (*R²* = 0.245, *p* <.001) and 31.2% of the variance in DSO (*R²* = 0.312, *p* <.001), indicating substantial explanatory power.

#### 3.3.1 Main effects, path differences, and interaction

**Figure 1:**
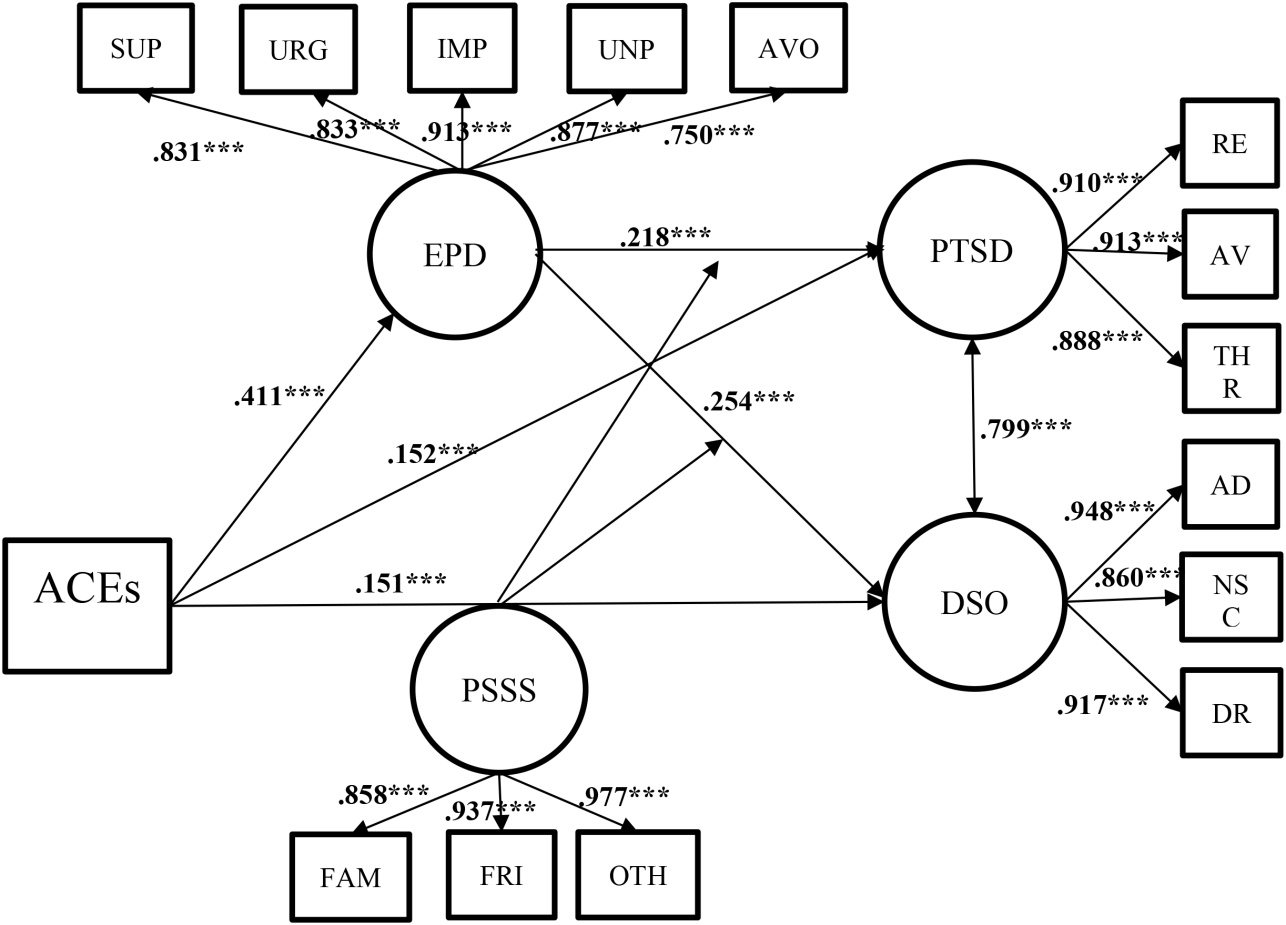
Standardized path coefficients for the full moderated mediation model. ACEs was modeled as an observed cumulative risk score and is enclosed in a rectangle. Values presented on the paths are standardized coefficients (β) derived from the final Latent Moderated Structural Equations (LMS) model. Solid black lines represent statistically significant structural paths, including the direct effects and the moderating effects originating from PSSS and pointing to the paths from EPD to PTSD/DSO (latent interactions). Non-significant main effects of PSSS on PTSD and DSO were estimated in the full model but are omitted from the diagram for visual clarity. Covariates (gender, age, and SES) were included in the model estimation but are also omitted for parsimony. ***p <.001. EPD, emotional processing deficits (indicators: SUP, suppression, URG, unregulated emotion, IMP, impoverished emotional experience, UNP, signs of unprocessed emotions, AVO, avoidance); PTSD, posttraumatic stress disorder symptoms (RE, re-experiencing, AV, avoidance; THR, sense of threat); DSO, disturbances in self-organization (AD, affective dysregulation; NSC, negative self-concept; DR, disturbed relationships); PSSS, perceived social support (FAM, family support; FRI, friend support; OTH, significant other support).

In the full model (**Table 2** and **Figure 1**), ACEs had significant direct positive effects on both PTSD (*b* = 0.086, 95% CI [0.061, 0.112], β = 0.152, *p* <.001) and DSO (*b* = 0.107, 95% CI [0.077, 0.137], β = 0.151, *p* <.001). To rigorously test H1, a Wald test was conducted on these unstandardized coefficients. Since PTSD and DSO were measured on the same psychometric scale within the ITQ, unstandardized comparisons are methodologically appropriate (17, 55). The test revealed that the unstandardized predictive association of ACEs with DSO was significantly stronger than that on PTSD (estimate of difference = 0.021, *p* = .035). However, it is crucial to note that the standardized effect sizes were virtually identical (β = 0.151 vs. 0.152). This indicates that while there is a statistically significant absolute difference on the psychometric scale, the relative magnitude of the associations is comparable. This suggests that cumulative trauma exerts practically parallel effects across both the core PTSD and DSO dimensions, rather than disproportionately impacting one over the other. Furthermore, the interaction between EPD and PSSS significantly predicted PTSD (*b* = -0.001, 95% CI [-0.003, -0.0001], β = -0.056, *p* = .015) and DSO (*b* = -0.002, 95% CI [-0.003, -0.0001], β = -0.053, *p* = .017), indicating that PSSS significantly moderated the relationships between EPD and both outcomes. The main effects of EPD on PTSD (β = 0.218, *p* <.001) and DSO (*β* = 0.254, *p* <.001) remained significant. Age and subjective SES significantly and negatively predicted EPD, while other covariates showed non-significant effects. A strong positive residual correlation was observed between PTSD and DSO (*r* = 0.799, *p* <.001).

**Table 2:** Path coefficients of the full moderated mediation model.

Path	Unstandardized (*b*)	*SE*	Standardized (β)	*p*	95% CI
Core Paths					
ACEs → EPD	2.13	0.074	0.411	<.001	[1.985, 2.274]
EPD → PTSD	0.024	0.002	0.218	<.001	[0.019, 0.029]
PSSS → PTSD	0.002	0.003	0.011	0.544	[-0.004, 0.008]
EPD × PSSS → PTSD	-0.001	0.001	-0.056	0.015	[-0.003, -0.0001]
ACEs → PTSD	0.086	0.013	0.152	<.001	[0.061, 0.112]
EPD → DSO	0.035	0.003	0.254	<.001	[0.029, 0.041]
PSSS → DSO	-0.002	0.004	-0.011	0.553	[-0.009, 0.005]
EPD × PSSS → DSO	-0.002	0.001	-0.053	0.017	[-0.003, -0.0001]
ACEs → DSO	0.107	0.015	0.151	<.001	[0.077, 0.137]

EPD × PSSS = latent interaction term of emotional processing deficits and perceived social support. Covariates (gender, age, SES) were included in the estimation. CI = confidence interval based on the Delta method.

#### 3.3.2 Conditional effects analysis

**Figure 2:**
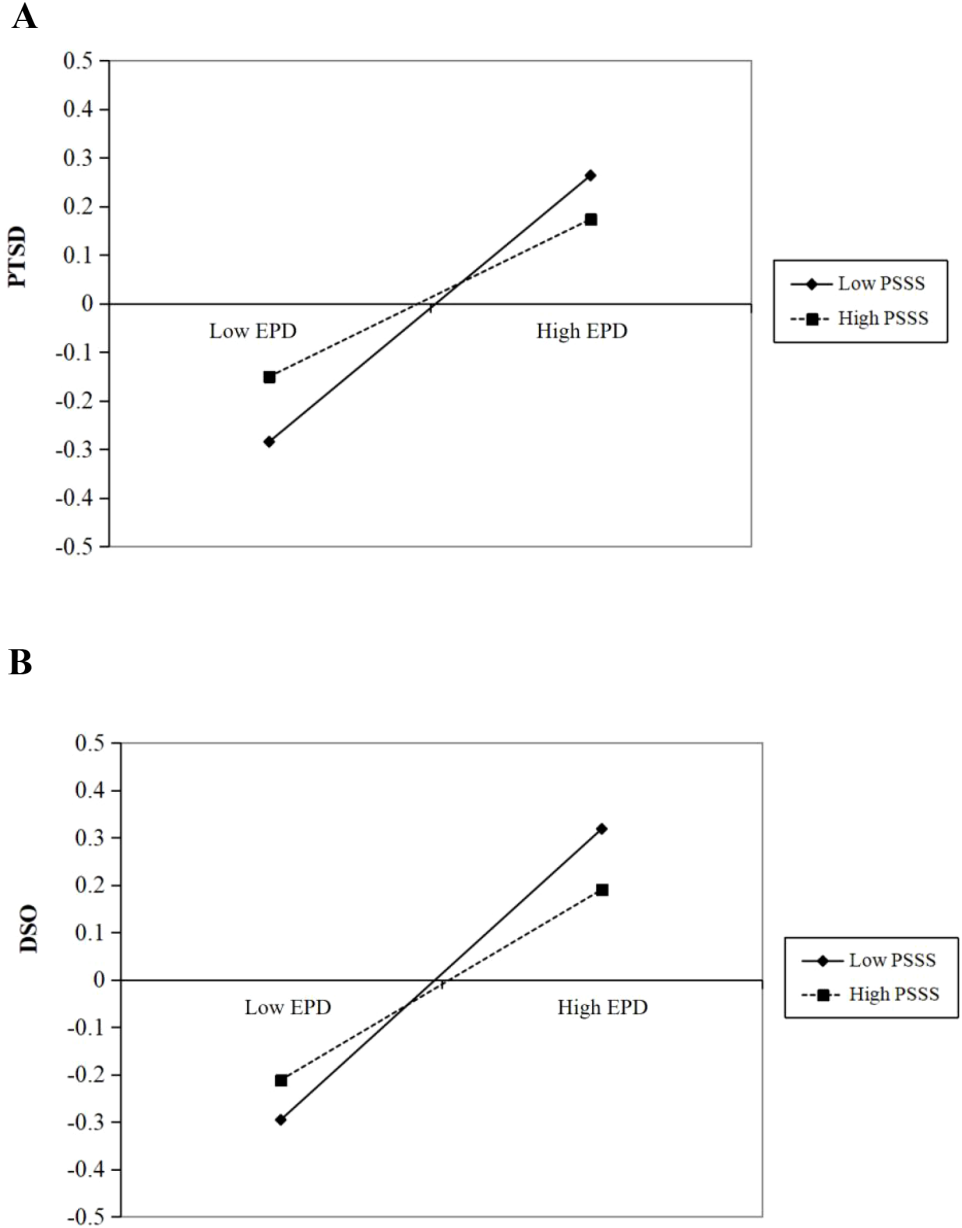
**(A)** Simple slopes of the interaction between emotional processing deficits (EPD) and perceived social support (PSSS) predicting PTSD symptoms.Low and high levels of PSSS are defined as one standard deviation below and above the mean, respectively. **(B)** Simple slopes of the interaction between emotional processing deficits (EPD) and perceived social support (PSSS) predicting DSO symptoms. Low and high levels of PSSS are defined as one standard deviation below and above the mean, respectively.

To further interpret the latent interactions, conditional effects of EPD on CPTSD dimensions were estimated at low (−1 *SD*), mean, and high (+1 *SD*) levels of perceived social support (see **Figure 2**). For PTSD symptoms, the association with EPD was significant at low PSSS (*b* = 0.030, *SE* = 0.003, *p* <.001), remained significant at the mean level of PSSS (*b* = 0.024, *SE* = 0.002, *p* <.001), and was significantly attenuated but remained positive at high PSSS (*b* = 0.018, *SE* = 0.004, *p* <.001). This pattern—where the effect size is robustly diminished at higher levels of support—indicates a significant buffering effect. A similar pattern was observed for DSO symptoms, where the conditional effect of EPD was significant at the mean level (*b* = 0.035, *SE* = 0.003, *p* <.001) and systematically attenuated at high levels of social support.

#### 3.3.3 Conditional indirect effects

The indirect effects of ACEs on symptoms via EPD were evaluated across PSSS levels (**Table 3**). For PTSD, the indirect effect was stronger in the low PSSS group (indirect effect = 0.064, β = 0.090) and remained significant but reduced in the high PSSS group (indirect effect = 0.038, β = 0.054). Similarly, for DSO, the indirect effect was stronger at low PSSS (0.089) compared to high PSSS (0.059). The index of moderated mediation was significant for both PTSD (Index = -0.003, 95% CI [-0.006, -0.001], *p* = .015) and DSO (Index = -0.004, 95% CI [-0.007, -0.001], *p* = .017). These results confirm that the mediating role of EPD in the ACE-symptom pathways is significantly moderated by perceived social support.

**Table 3:** Conditional indirect effects and index of moderated mediation.

Outcome	PSSS Level	Indirect Effect	*SE*	*p*	95% CI
PTSD	Low (-1 SD)	0.064	0.008	<.001	[0.049, 0.079]
	High (+1 SD)	0.038	0.008	<.001	[0.022, 0.053]
	Index of mod. med.	-0.003	0.001	0.015	[-0.006, -0.001]
DSO	Low (-1 SD)	0.089	0.009	<.001	[0.071, 0.107]
	High (+1 SD)	0.059	0.01	<.001	[0.040, 0.078]
	Index of mod. med.	-0.004	0.002	0.017	[-0.007, -0.001]

Indirect effect represents the impact of ACEs on the outcome variables via EPD. The index of moderated mediation represents the change in the indirect effect for every one-unit increase in PSSS. CI, confidence interval.

### 3.4 Sensitivity and exploratory analyses

Given the zero-inflated nature of the full sample, we re-estimated the full LMS model exclusively among participants who endorsed experiencing a traumatic event on the ITQ (*n* = 1, 643). While the main pathways (ACEs → EPD → CPTSD dimensions) remained highly significant, the general buffering effect of overall PSSS was largely attenuated for both PTSD (*p* = .705) and DSO (*p* = .180) (see **Supplementary Table 1**).

To further unpack this nullification and explore whether specific sources of support functioned differently, we conducted source-specific moderation analyses (Family, Friends, and Significant Others) across both samples (**Supplementary Table 2**). In the highly powered full sample (*N* = 5, 771), all three sources demonstrated significant buffering effects (*p*s <.05). However, in the trauma-exposed subsample, the buffering capacities of both family and significant others largely disappeared (all *p*s >.10). Strikingly, only *friend support* retained a marginally significant buffering trend specifically against DSO symptoms (*p* = .073). These findings highlight critical boundary conditions for the efficacy of social support depending on the source and trauma history.

## 4 Discussion

Consistent with developmental trauma literature (28), emotional processing deficits significantly mediated the relationship between ACEs and CPTSD symptoms. Chronic exposure to uncontrollable stressors during critical developmental periods frequently overwhelms a child’s maturing emotion regulation systems (3, 4).

Our results indicate that EPD serves as a core mediating factor associated with the pathway from early trauma into current psychopathology (17). Viewing this through the predictive processing framework (62), these severe emotional processing deficits can be understood as “pathological predictive errors”—trauma-induced, rigid prior predictions that fail to update in safe environments. When encountering current stressors, traumatized individuals with profound EPD are unable to adaptively process negative emotions, resorting instead to experiential avoidance or emotional suppression. This chronic dysregulation may exacerbate traditional hypervigilance (63) but directly spawns the affective instability and negative self-concept characterizing DSO. This finding also well explains the “parallel destruction” observed across both dimensions in the standardized scores, confirming that DSO symptoms essentially reflect a pervasive extension of emotional processing deficits into the self-system (37, 64).

A theoretically relevant finding is the significant moderating role of social support in the latter stage of the mediation process. Although EPD invariably predicted more severe CPTSD symptoms, this toxic association was significantly attenuated among individuals reporting high social support.

While our findings in the full sample align with the classic stress-buffering hypothesis (41), indicating that PSSS provides a ‘safe base’ for co-regulation, it is essential to interpret this moderating effect with nuance. Given the large sample size, the standardized interaction effect was small (β ≈ -0.05), suggesting a modest rather than absolute buffering role. Importantly, the present findings should be interpreted specifically in terms of emotional processing deficits as operationalized by the EPS-15 facets, rather than broader constructs of emotion dysregulation. Furthermore, the conditional effects analysis suggests that the buffering effect of perceived social support operates across the full observed range, rather than being restricted to a specific threshold, which is consistent with a general attenuation rather than a region-specific effect.

Notably, these findings may help reconcile previously inconsistent findings regarding the buffering versus reverse-buffering effects of social support. While a general buffering effect was observed in the overall sample, this effect was attenuated and became non-significant in the trauma-exposed subsample, and family support no longer demonstrated a protective role. This pattern is consistent with the notion of a reverse-buffering effect and suggests that, under certain conditions—particularly when the source of support is closely tied to early adverse experiences (45, 46)—social support may fail to function as a protective factor or may even become maladaptive. This may be related to heightened interpersonal sensitivity, mistrust, or the reactivation of trauma-related schemas in close relationships. In contrast, friend support showed a marginal buffering trend against DSO symptoms in the trauma-exposed group, suggesting that peer relationships may serve as a relatively more adaptive source of support in this developmental context (65, 66). Taken together, these results indicate that the buffering effect of social support is conditional rather than universal, depending on both the individual’s trauma history and the source of support. This perspective provides a potential theoretical explanation for previously conflicting findings, supporting a conditional buffering framework wherein the protective role of social support fundamentally depends on the individual’s trauma history and the relational context of that support. Future research is needed to further delineate the conditions under which social support may shift from protective to maladaptive.

Clinically, because EPD functions as a transdiagnostic mechanism, trauma-informed interventions may benefit from incorporating components targeting emotional regulation capacities prior to trauma-focused exposure work, such as the Skills Training in Affective and Interpersonal Regulation (STAIR) protocol (67–69). Furthermore, interventions must extend to systemic levels. Given the buffering effect of social support, clinicians should actively assist traumatized individuals in rebuilding their social networks (70).

## 5 Limitations

Several important limitations must be acknowledged. First, the cross-sectional design limits definitive causal inference, particularly regarding the dynamic relationships between emotional processing deficits and CPTSD symptoms. However, it is important to consider the inherent temporal ordering of key variables in the present study. Adverse childhood experiences, by definition, occur prior to the age of 18, whereas emotional processing deficits and CPTSD symptoms reflect current functioning in emerging adulthood. This temporal asymmetry provides a theoretically plausible basis for modeling ACEs as antecedent variables, even within a cross-sectional framework. Nevertheless, the second stage of the mediation process (i.e., from emotional processing deficits to CPTSD symptoms) would benefit from longitudinal designs to more rigorously establish directionality. Future research employing prospective or multi-wave designs is warranted to further validate these pathways. Second, our data distributions exhibited substantial zero-inflation and floor effects, as the vast majority of our non-clinical university sample reported neither trauma exposure nor clinically relevant symptoms (e.g., CPTSD prevalence was 1.2%). Consequently, our model primarily captures variations in *subclinical* symptomatology rather than disorder-level psychopathology. Third, our reliance on a massive sample size *(N* = 5, 771) provided immense statistical power, which inevitably rendered even modest effect sizes highly significant (*p* <.01). Furthermore, the high internal consistency observed for the ITQ and PSSS scales may reflect item redundancy, which could potentially inflate associations in single-source designs, particularly for interaction effects. Fourth, regarding model fit, global fit indices are not fully available or directly interpretable in LMS models with latent interactions; therefore, model evaluation had to rely on a combination of baseline fit and log-likelihood comparisons. Notably, the SRMR in our baseline model was elevated (0.124). Although simulation studies indicate SRMR can be inflated in models with highly skewed outcomes and multiple exogenous variables (59), this remains a specification limitation. Fifth, relying exclusively on self-report measures introduces the risk of common method bias. Although Harman’s single-factor test suggested it was not the primary driver of variance, this test is not diagnostic enough to substantially rule out method bias (53). Finally, while we utilized a cumulative risk approach for ACEs, exploring specific dimensions of adversity (e.g., threat vs. deprivation) might reveal unique mechanistic pathways to DSO (71).

## 6 Conclusion

In conclusion, this study indicates that while adverse childhood experiences are significantly associated with complex psychopathology dimensions via emotional processing deficits, social support may serve as a modest buffering mechanism to help attenuate this positive association. Importantly, however, our findings highlight that the protective efficacy of social support is not universal; rather, it is highly constrained by the individual’s trauma history and the specific source of support. For emerging adults with a history of early adversity, family support may largely lose its buffering capacity, underscoring the vital, compensatory importance of peer and friendship networks in trauma recovery.

## Data Availability

The raw data supporting the conclusions of this article will be made available by the authors, without undue reservation.

## References

[1] Felitti VJ Anda RF Nordenberg D Williamson DF Spitz AM Edwards V . Relationship of childhood abuse and household dysfunction to many of the leading causes of death in adults: The Adverse Childhood Experiences (ACE) Study. Am J Prev Med. (1998) 14:245–58. doi: 10.1016/S0749-3797(98)00017-8. PMID: 9635069

[2] Hughes K Bellis MA Hardcastle KA Sethi D Butchart A Mikton C . The effect of multiple adverse childhood experiences on health: a systematic review and meta-analysis. Lancet Public Health. (2017) 2:e356–66. doi: 10.1016/S2468-2667(17)30118-4. PMID: 29253477

[3] Teicher MH Samson JA Anderson CM Ohashi K . The effects of childhood maltreatment on brain structure, function and connectivity. Nat Rev Neurosci. (2016) 17:652–66. doi: 10.1038/nrn.2016.111. PMID: 27640984

[4] Anda RF Felitti VJ Bremner JD Walker JD Whitfield C Perry BD . The enduring effects of abuse and related adverse experiences in childhood: A convergence of evidence from neurobiology and epidemiology. Eur Arch Psychiatry Clin Neurosci. (2006) 256:174–86. doi: 10.1007/s00406-005-0624-4. PMID: 16311898 PMC3232061

[5] Kessler RC McLaughlin KA Green JG Gruber MJ Sampson NA Zaslavsky AM . Childhood adversities and adult psychopathology in the WHO World Mental Health Surveys. Br J Psychiatry. (2010) 197:378–85. doi: 10.1192/bjp.bp.110.080499. PMID: 21037215 PMC2966503

[6] McLaughlin KA Colich HL Levenson AR Fassberg EA . Childhood adversity and adult psychopathology. Annu Rev Clin Psychol. (2020) 16:277–312.

[7] Li Y He Z Liu Z Peng N Xiao Y Ye Y . Adverse childhood experiences and the diagnosis of ICD-11 post-traumatic stress disorder or complex post-traumatic stress disorder: A systematic review and three-level meta-analysis. Trauma Violence Abuse. (2025) 26:1–15. doi: 10.1177/15248380251336188. PMID: 40317203

[8] Courtois CA Ford JD . Treatment of complex trauma: A sequenced, relationship-based approach. New York, NY: Guilford Press (2013).

[9] Cloitre M Stolbach BC Herman JL van der Kolk B Pynoos R Wang J . A developmental approach to complex PTSD: Childhood and adult cumulative trauma as predictors of symptom complexity. J Traumatic Stress. (2009) 22:399–408. doi: 10.1002/jts.20444. PMID: 19795402

[10] Herman JL . Trauma and recovery. New York, NY: Basic Books (1992).

[11] Cloitre M Garvert DW Brewin CR Bryant RA Maercker A . Evidence for proposed ICD-11 PTSD and complex PTSD: A latent profile analysis. Eur J Psychotraumatol. (2013) 4:20706. doi: 10.3402/ejpt.v4i0.20706. PMID: 23687563 PMC3656217

[12] Maercker A Brewin CR Bryant RA Cloitre M van Ommeren M Jones LM . Diagnosis and classification of disorders specifically associated with stress: proposals for ICD-11. World Psychiatry. (2013) 12:198–206. doi: 10.1002/wps.20050. PMID: 24096776 PMC3799241

[13] Brewin CR . Complex post-traumatic stress disorder: A new diagnosis in ICD-11. BJPsych Adv. (2020) 26:145–52. doi: 10.1192/bja.2019.48. PMID: 38385431

[14] Brewin CR Cloitre M Hyland P Shevlin M Maercker A Bryant RA . A review of current evidence regarding the ICD-11 proposals for diagnosing PTSD and complex PTSD. Clin Psychol Rev. (2017) 58:1–15. doi: 10.1016/j.cpr.2017.09.001. PMID: 29029837

[15] World Health Organization . International classification of diseases for mortality and morbidity statistics (11th Revision) (2018). Available online at: https://icd.who.int/browse11/l-m/en (Accessed March 15, 2026).

[16] D’Andrea W Ford J Bradley B Pelcovitz D van der Kolk BA . Understanding interpersonal trauma in children: why we need a developmentally appropriate trauma diagnosis. Am J Orthopsychiatry. (2012) 82:187–200. doi: 10.1111/j.1939-0025.2012.01154.x. PMID: 22506521

[17] Cloitre M Shevlin M Brewin CR Bisson JI Roberts NP Maercker A . The International Trauma Questionnaire: development of a self-report measure of ICD-11 PTSD and complex PTSD. Acta Psychiatrica Scandinavica. (2018) 138:536–46. doi: 10.1111/acps.12956. PMID: 30178492

[18] Karatzias T Shevlin M Fyvie C Hyland P Efthymiadou E Wilson D . Evidence of distinct profiles of Posttraumatic Stress Disorder (PTSD) and Complex Posttraumatic Stress Disorder (CPTSD) based on the new ICD-11 Trauma Questionnaire (ICDTQ). J Affect Disord. (2017) 207:181–7. doi: 10.1016/j.jad.2016.09.032. PMID: 27723542

[19] Ben-Ezra M Karatzias T Hyland P Brewin CR Cloitre M Bisson JI . Posttraumatic stress disorder (PTSD) and complex PTSD (CPTSD) as per ICD-11 proposals: A population study in Israel. Depression Anxiety. (2018) 35:264–74. doi: 10.1002/da.22723. PMID: 29451956

[20] Ho GWK Chan KL Karatzias T Hyland P Fung HW Shevlin M . Prevalence and validity of ICD-11 posttraumatic stress disorder (PTSD) and complex PTSD: A population-based survey of Hong Kong adults. Asian J Psychiatry. (2024) 96:104045. doi: 10.1016/j.ajp.2024.104045. PMID: 38643682

[21] Karatzias T Hyland P Bradley A Cloitre M Roberts NP Bisson JI . Risk factors and comorbidity of ICD-11 PTSD and complex PTSD: Findings from a trauma-exposed population based sample of adults in the United Kingdom. Depression Anxiety. (2019) 36:887–94. doi: 10.1002/da.22934. PMID: 31268218

[22] Li S Guo C Chan SSS . ICD-11 posttraumatic stress disorder and complex PTSD among hospital medical workers in China: impacts of wenchuan earthquake exposure, workplaces, and sociodemographic factors. Front Psychiatry. (2022) 12:735861. doi: 10.3389/fpsyt.2021.735861. PMID: 35111084 PMC8801437

[23] Ren Y Yang S Peng Y Liu A Zhu Z . Retrospective ACEs predict complex PTSD symptoms in a large sample of Chinese young adults longitudinally: The moderating role of self-compassion. BMC Psychiatry. (2024) 24:425. doi: 10.1186/s12888-024-05830-z. PMID: 38844888 PMC11155039

[24] Hyland P Murphy J Shevlin M Vallières F McElroy E Elklit A . Variation in post-traumatic response: the role of trauma type in predicting ICD-11 PTSD and CPTSD symptoms. Soc Psychiatry Psychiatr Epidemiol. (2017) 52:727–36. doi: 10.1007/s00127-017-1350-8. PMID: 28194504

[25] Harris J Loth E Sethna V . Tracing the paths: A systematic review of mediators of complex trauma and complex post-traumatic stress disorder. Front Psychiatry. (2024) 15:1331256. doi: 10.3389/fpsyt.2024.1331256. PMID: 38510809 PMC10951104

[26] Ford JD . Complex PTSD: research directions for nosology/assessment, treatment, and public health. Eur J Psychotraumatol. (2015) 6:27584. doi: 10.3402/ejpt.v6.27584. PMID: 25994023 PMC4439420

[27] Bloomfield MAP Chang T Wood MJ Lyons LM Cheng Z Bauer-Staeb C . Psychological processes mediating the association between developmental trauma and specific psychotic symptoms in adults: A systematic review and meta-analysis. World Psychiatry. (2021) 20:107–23. doi: 10.1002/wps.20841. PMID: 33432756 PMC7801841

[28] Guo T Huang L Hall DL Jiao C Chen ST Yu Q . The relationship between childhood adversities and complex posttraumatic stress symptoms: a multiple mediation model. Eur J Psychotraumatol. (2021) 12:1936921. doi: 10.1080/20008198.2021.1936921. PMID: 34249246 PMC8245101

[29] Kampling H Kruse J Lampe A Nolte T Hettich N Brähler E . Epistemic trust and personality functioning mediate the association between adverse childhood experiences and posttraumatic stress disorder and complex posttraumatic stress disorder in adulthood. Front Psychiatry. (2022) 13:919191. doi: 10.3389/fpsyt.2022.919191. PMID: 36032256 PMC9399466

[30] Ehring T Quigley L . Emotion regulation and trauma. In: Emotion regulation and psychopathology: A transdiagnostic approach to etiology and treatment. New York, NY: Guilford Press (2010). p. 165–89.

[31] Gross JJ . Emotion regulation: Current status and future prospects. psychol Inq. (2015) 26:1–26. doi: 10.1080/1047840X.2014.940781. PMID: 41909888

[32] Gratz KL Roemer L . Multidimensional assessment of emotion regulation and dysregulation: Development, factor structure, and initial validation of the difficulties in emotion regulation scale. J Psychopathol Behav Assess. (2004) 26:41–54. doi: 10.1023/B:JOBA.0000007455.08539.94. PMID: 37215487

[33] Maroti D Axelsson E Ljótsson B Andersson G Lumley MA Johansson R . Psychometric properties of the emotional processing scale in individuals with psychiatric symptoms and the development of a brief 15-item version. Sci Rep. (2022) 12:10456. doi: 10.1038/s41598-022-14712-x. PMID: 35729355 PMC9213431

[34] Teicher MH Samson JA . Annual research review: Enduring neurobiological effects of childhood abuse and neglect. J Child Psychol Psychiatry. (2016) 57:241–66. doi: 10.1111/jcpp.12507. PMID: 26831814 PMC4760853

[35] Uzun K Tagay Ö Cırcır O . Childhood traumas and future expectations in adolescents: Examination the role of perceived social support and attitudes to seeking psychological help. Curr Psychol. (2024) 43:7116–30. doi: 10.1007/s12144-023-04925-2. PMID: 41933263

[36] Ehlers A Clark DM . A cognitive model of posttraumatic stress disorder. Behav Res Ther. (2000) 38:319–45. doi: 10.1016/S0005-7967(99)00123-0. PMID: 10761279

[37] Karatzias T Shevlin M Hyland P Brewin CR Cloitre M Bradley A . The role of negative cognitions, emotion regulation strategies, and attachment style in complex post-traumatic stress disorder: Implications for new and existing therapies. Br J Clin Psychol. (2018) 57:177–85. doi: 10.1111/bjc.12172. PMID: 29355986

[38] Jeong Y . The relationship between childhood trauma and suicidal ideation among Korean older adults: mediating role of post-traumatic growth and moderated mediation effect of social support. Curr Psychol. (2025) 44:18164–74. doi: 10.1007/s12144-025-08472-w. PMID: 41933263

[39] Southwick SM Vythilingam M Charney DS . The psychobiology of depression and resilience to stress: Implications for prevention and treatment. Annu Rev Clin Psychol. (2005) 1:255–91. doi: 10.1146/annurev.clinpsy.1.102803.143948. PMID: 17716089

[40] Bonanno GA . Loss, trauma, and human resilience: Have we underestimated the human capacity to thrive after extremely aversive events? Am Psychol. (2004) 59:20–8. doi: 10.1037/0003-066X.59.1.20. PMID: 14736317

[41] Cohen S Wills TA . Stress, social support, and the buffering hypothesis. psychol Bull. (1985) 98:310–37. doi: 10.1037/0033-2909.98.2.310. PMID: 3901065

[42] Thoits PA . Mechanisms linking social ties and support to physical and mental health. J Health Soc Behav. (2011) 52:145–61. doi: 10.1177/0022146510395592. PMID: 21673143

[43] Cobb S . Social support as a moderator of life stress. Psychosomatic Med. (1976) 38:300–14. doi: 10.1097/00006842-197609000-00003. PMID: 981490

[44] Ozbay F Johnson DC Southwick SM Morgan CA Charney DS Holahan CK . Social support and resilience to stress: From neurobiology to clinical practice. Psychiatry (Edgmont). (2007) 4:35–40. PMC2921311 20806028

[45] Lou X Hu H Zou X Chen X Wu Y Zhang T . Adverse childhood experiences and family health in adulthood: is perceived social support an accelerant or a buffer? J Interpersonal Violence. (2025), 08862605251343193. doi: 10.1177/08862605251343193. PMID: 40474766

[46] Zhou K Zhu X Lu C Yang Y Chen S . Adverse childhood experiences, basic psychological needs, and adolescent affective distress: Revisiting the buffering role of resilience factors. J Res Adolescence. (2025) 35:e70080. doi: 10.1111/jora.70080. PMID: 41058052 PMC12504805

[47] Kaniasty K Norris FH . Longitudinal linkages between perceived social support and posttraumatic stress symptoms: Sequential roles of social causation and social selection. J Traumatic Stress. (2008) 21:274–81. doi: 10.1002/jts.20334. PMID: 18553415

[48] Ho GWK Karatzias T Armour C Chien WT Wong WK Shevlin M . The Chinese version of the Adverse Childhood Experiences-International Questionnaire (ACE-IQ): Reliability and validity. Child Abuse Negl. (2019) 93:172–82. doi: 10.1016/j.chiabu.2019.04.017. PMID: 31108407

[49] Xu H Wang Y Li C Liang A Cai Y . Reliability and validity of the Chinese version of the Adverse Childhood Experiences-International Questionnaire among men who have sex with men. Shanghai J Prev Med. (2025) 37:878–83. doi: 10.19428/j.cnki.sjpm.2025.24845

[50] Zimet GD Dahlem NW Zimet SG Farley GK . The multidimensional scale of perceived social support. J Pers Assess. (1988) 52:30–41. doi: 10.1207/s15327752jpa5201_2. PMID: 2280326

[51] Zhao Q Su L Wang X Du J Peng X . The performance evaluation of Perceived Social Support Scale (PSSS) in the front-line staff. Adv Psychol. (2022) 12:952–61. doi: 10.12677/ap.2022.123113

[52] IBM Corp . IBM SPSS Statistics for Windows, Version 26.0. Armonk, NY: IBM Corp (2019).

[53] Podsakoff PM MacKenzie SB Lee JY Podsakoff NP . Common method biases in behavioral research: A critical review of the literature and recommended remedies. J Appl Psychol. (2003) 88:879–903. doi: 10.1037/0021-9010.88.5.879. PMID: 14516251

[54] Muthén LK Muthén BO . Mplus User’s Guide. Los Angeles, CA: Muthén & Muthén (1998–2017).

[55] Kline RB . Principles and practice of structural equation modeling. New York, NY: Guilford publications (2015).

[56] Yuan KH Bentler PM . Three likelihood-based methods for mean and covariance structure analysis with nonnormal data. Sociological Method. (2000) 30:165–200. doi: 10.1111/0081-1750.00078. PMID: 41940437

[57] Klein A Moosbrugger H . Maximum likelihood estimation of latent interaction effects with the latent moderated structural equations method. Struct Equation Modeling: A Multidiscip J. (2000) 7:457–74. doi: 10.1207/S15328007SEM0704_6. PMID: 38329648

[58] Little TD Bovaird JA Widaman KF . On the merits of orthogonalizing powered and product terms: Implications for modeling interactions among latent variables. Struct Equation Modeling: A Multidiscip J. (2006) 13:497–519. doi: 10.1207/s15328007sem1304_1. PMID: 40931341

[59] Maslowsky J Jager J Hemken D . Estimating and interpreting latent variable interactions: A tutorial for applying the latent moderated structural equations method. Int J Behav Dev. (2015) 39:87–96. doi: 10.1177/0165025414552301. PMID: 26478643 PMC4606468

[60] Hayes AF . Introduction to mediation, moderation, and conditional process analysis: A regression-based approach. New York, NY: Guilford Press (2017).

[61] Fornell C Larcker DF . Evaluating structural equation models with unobservable variables and measurement error. J Marketing Res. (1981) 18:39–50. doi: 10.1177/002224378101800104. PMID: 41930703

[62] Putica A Agathos J . Reconceptualizing complex posttraumatic stress disorder: A predictive processing framework for mechanisms and intervention. Neurosci Biobehav Rev. (2024) 164:105836. doi: 10.1016/j.neubiorev.2024.105836. PMID: 39084584

[63] Foa EB Kozak MJ . Emotional processing of fear: Exposure to corrective information. psychol Bull. (1986) 99:20–35. doi: 10.1037/0033-2909.99.1.20. PMID: 2871574

[64] Li J Wang W Zhang W Qu Z . The impact of cumulative trauma on complex posttraumatic stress disorder in children: The mediating role of experiential avoidance and negative cognitive emotion regulation strategies. psychol Dev Educ. (2022) 38:683–91. doi: 10.16187/j.cnki.issn1001-4918.2022.05.09

[65] Borowski SK Zeman JL . Emotion socialization within adolescent friendships: Considering the role of friends’ emotion regulation, perceptions of friends, and expectations of support. J Res Adolescence. (2025) 35:e70044. doi: 10.1111/jora.70044. PMID: 40671270 PMC12267931

[66] Todorovic L Bozhar H de Rooij SR Bogaerts A Boyer BE Larsen H . Bidirectional associations of problematic social media use and problematic gaming with mental health difficulties and strengths in adolescents: Sex and social support as potential moderators. J Res Adolescence. (2025) 35:e70076. doi: 10.1111/jora.70076. PMID: 40944323 PMC12432349

[67] Fonagy P Allison E . The role of mentalizing and epistemic trust in the therapeutic relationship. Psychotherapy. (2014) 51:372–80. doi: 10.1037/a0036505. PMID: 24773092

[68] Cloitre M Courtois CA Charuvastra A Carapezza R Stolbach BC Green BL . Treatment of complex PTSD: Results of the ISTSS expert clinician survey on best practices. J Traumatic Stress. (2010) 23:615–27. doi: 10.1002/jts.20584. PMID: 22147449

[69] Cloitre M Cohen LR Koenen KC . Treating survivors of childhood abuse: Psychotherapy for the interrupted life. New York, NY: Guilford Press (2006).

[70] Ozer EJ Best SR Lipsey TL Weiss DS . Predictors of posttraumatic stress disorder and symptoms in adults: A meta-analysis. psychol Bull. (2003) 129:52–73. doi: 10.1037/0033-2909.129.1.52. PMID: 12555794

[71] McLaughlin KA Sheridan MA Lambert HK . Childhood adversity and neural development: Deprivation and threat as distinct dimensions of early experience. Neurosci Biobehav Rev. (2014) 47:578–91. doi: 10.1016/j.neubiorev.2014.10.012. PMID: 25454359 PMC4308474

