# Supplementary material for: Emotional processing deficits mediate the association between adverse childhood experiences and CPTSD symptoms: the moderating role of perceived social support
Source: Front Psychiatry. 2026 Apr 28;17:1834482. doi: 10.3389/fpsyt.2026.1834482 (PMC13161147; doi:10.3389/fpsyt.2026.1834482)
Supplement: Supplementary file 1 [file DataSheet1.pdf]

Supplementary Material

Supplementary Table 1. Path coefficients of the latent moderated mediation model in the trauma-exposed subsample (n = 1,643).

Path	Unstandardized (b)	*SE*	Standardized (β)	p
**First Stage Mediation**				
ACEs → EPD	1.721	0.110	0.407***	< .001
**Paths to PTSD Symptoms**				
EPD → PTSD	0.062	0.007	0.333***	< .001
PSSS (General) → PTSD	-0.027	0.009	-0.094**	.003
EPD × PSSS → PTSD	0.001	0.002	0.013	.705
ACEs → PTSD	0.051	0.025	0.065*	.043
**Paths to DSO Symptoms**				
EPD → DSO	0.086	0.008	0.391***	< .001
PSSS (General) → DSO	-0.055	0.012	-0.158***	< .001
EPD × PSSS → DSO	0.002	0.002	0.044	.180
ACEs → DSO	0.026	0.029	0.028	.365

Note. ACEs = Adverse Childhood Experiences; EPD = Emotional Processing Deficits; PSSS = Perceived Social Support; PTSD = Post-Traumatic Stress Disorder core symptoms; DSO = Disturbances in Self-Organization. EPD × PSSS represents the latent interaction term. Covariates (gender, age, and subjective SES) were included in the model estimation but are omitted from the table for parsimony. The trauma-exposed

subsample (n = 1,643) consists of participants who explicitly reported experiencing a traumatic event on the International Trauma Questionnaire (ITQ). *p < .05. **p < .01. ***p < .001.

Supplementary Table 2. Summary of source-specific moderation effects (Interaction terms) across the full sample and trauma-exposed subsample.

Interaction Path (Moderation)	Full Sample (N = 5,771) p-value	Trauma Subsample (n = 1,643) p-value
**Family Support**		
EPD × Family → PTSD	.005**	.650
EPD × Family → DSO	.002**	.243
**Friend Support**		
EPD × Friends → PTSD	.019*	.662
EPD × Friends → DSO	.034*	.073†
**Significant Others**		
EPD × Others → PTSD	.039*	.579
EPD × Others → DSO	.045*	.135

Note. Models in Table 2 utilized observed variable interactions for support sources to ensure convergence. All models controlled for gender, age, and subjective SES. The trauma-exposed subsample (n = 1,643) consists of participants who explicitly reported experiencing a traumatic event on the International Trauma Questionnaire (ITQ). The contrast highlights the complete nullification of family and significant other support, alongside the unique marginal retention of friend support buffering against DSO in the trauma-exposed cohort. †p < .10 (marginally significant). *p < .05. **p < .01.
